# Supplementary material for: Complex effects of chytrid parasites on the growth of the cyanobacterium Planktothrix rubescens across interacting temperature and light gradients
Source: ISME Commun. 2022 Sep 30;2:93. doi: 10.1038/s43705-022-00178-5 (PMC9723700; doi:10.1038/s43705-022-00178-5)
Supplement: Supplementary file 1 — Supplementary material [file 43705_2022_178_MOESM1_ESM.docx]

# **Supplementary material:** **Complex effects of chytrid parasites on the growth of the cyanobacterium *Planktothrix rubescens* across interacting temperature and light gradients**

# *I. Experimental design:*


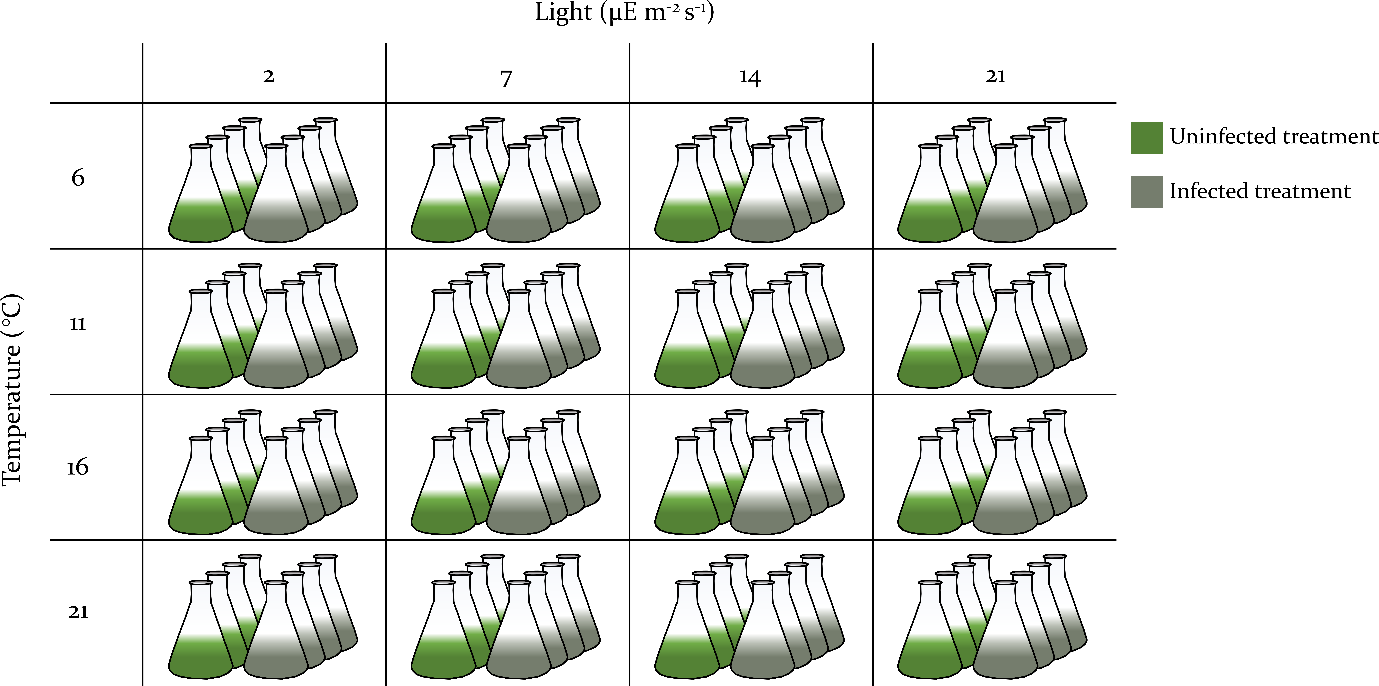


**Fig. S1**. Experimental setup indicating the factorial combination of temperature, light & infection levels, as well as the 4 replicates per treatment.

## *II. Model parameter dependence on temperature and light*

The infectivity parameter *I* is calculated as exponential decay of $H_{u}$ between *t_0_* and at *t* days (where t = 2). *Z* at *t_0_* is the initial zoospore concentration:

$$I=-\frac{ln\left[ \frac{H_{u}\left( t \right)}{H_{u}\left( t_{0} \right)} \right]}{Z\left( t_{0} \right) t}$$

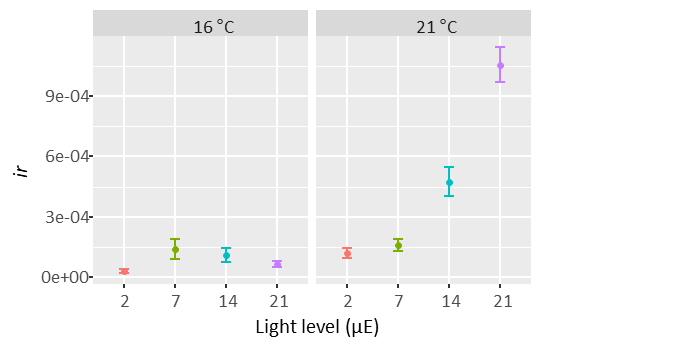
Fig. S2 illustrates how *I* changes with temperature and light, based on these calculations:

**Fig. S2**. Light and temperature-dependence of the infectivity parameter I

*I*

Light (μE m^-2^ s^-1^)

The production of zoospores *p* per nL bio-volume was calculated from supplementary data from Frenken et al. (2020). It is calculated from infected *P. rubescens* cultures (NIVA-CYA97/1). Production is the number of new zoospores divided by the bio-volume 2 days before. The zoospore production was calculated between days 4 to 10 when the infection is established, and prevalence of infection is still increasing.


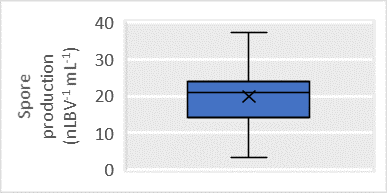


**Fig. S3**. Spore production per biovolume (nL mL^-1^)

The model incorporates exponentially increasing or decreasing parameters with temperature for the development time of zoospores 𝜏, mortality rate of zoospores *m_z_*, and the mortality rate of uninfected and infected host, *m_u_* and *m_i_*. according to the following equation:

$$y=a\cdot e^{b\cdot T}$$

where *T* is temperature and *y* is the focal parameter.

Development time of zoospores 𝜏 has been set to 2 days at 21 degrees and 3 days at 16.
*a* is 16.42, *b* is -0.099.

Mortality rate of the uninfected host *m_u_* has been fitted to 1 percent of the respective growth rate.
*a* is 4.62e-04, *b* is 7.83e-02.

Mortality rate of the infected host *m_i_* is, like m_u_, based on an exponential fit through the 1 percent growth rate, with an increase in the dependence on temperature of 50 percent, and a higher initial mortality rate.
*a* is 3.05e-02, *b* is 1.17e-01.

The life-time of zoospores was determined to be 2.71 days at 17 °C, which translates into a mortality rate *m_z_* of 0.36. subsequently the *m_z_* was set at 0.05, 0.12, and 0.5 for 6 °C, 11 °C and 21 °C respectively.
*a* is 0.035, *b* is 0.128.
